# Multifaceted Roles of Adipose Tissue-Derived Exosomes in Physiological and Pathological Conditions

**DOI:** 10.3389/fphys.2021.669429

**Published:** 2021-04-20

**Authors:** Yunnan Liu, Chen Wang, Mengying Wei, Guodong Yang, Lijun Yuan

**Affiliations:** ^1^Department of Ultrasound Diagnostics, Tangdu Hospital, Fourth Military Medical University, Xi’an, China; ^2^State Key Laboratory of Cancer Biology, Department of Biochemistry and Molecular Biology, Fourth Military Medical University, Xi’an, China

**Keywords:** adipose tissue, exosomes, clinical application, physiological functions, pathological functions

## Abstract

Adipose tissue functions importantly in the bodily homeostasis and systemic metabolism, while obesity links to multiple disorders. Beyond the canonical hormones, growth factors and cytokines, exosomes have been identified to play important roles in transmission of information from adipose tissue to other organs. Exosomes are nanoscale membrane vesicles secreted by donor cells, and transfer the genetic information to the recipient cells where the encapsulated nucleic acids and proteins are released. In this review, we elaborate the recent advances in the biogenesis and profiling of adipose tissue derived exosomes, and their physiological and pathological effects on different organs. Moreover, the potential significance of the exosomes as therapeutic vehicles or drugs is also discussed.

## Introduction

In mammals, there are two main types of adipose tissues termed as white adipose tissue (WAT) and brown adipose tissue (BAT), respectively. WAT and BAT function significantly different (Zwick et al., 2018). WAT stores excess energy in the form of triglycerides (van Eenige et al., 2018), while BAT improves the energy expenditure by activating uncoupling protein 1 (UCP1) (Oelkrug et al., 2015). In WAT triglycerides (TAG) and other neutral lipids are stored in large lipid droplets. Hydrolysis of TAGs can provide substrates to meet systemic metabolic needs during negative energy balance (Scherer, 2006). WAT can be further divided into visceral adipose tissue (VAT) and subcutaneous adipose tissue (SAT) (Barreira et al., 2011), mainly distributed in the chest and abdominal cavity (Britton et al., 2013). Besides the commonly studied WAT and BAT, recent studies have identified beige adipose tissue, which is more closely related to WAT in development and similar to BAT in shape and function (Kaisanlahti and Glumoff, 2019). Beige adipose cells can also dissipate energy to fight obesity through uncoupled mitochondrial respiration (Cypess and Kahn, 2010; Nedergaard et al., 2011; Chen and Pfeifer, 2017).

Normal VAT functions importantly in maintaining normal metabolism, while excessive VAT is a risk factor for metabolic diseases (Fox et al., 2007). Previous studies have shown that visceral adipocytes promote systemic inflammation by secreting cytokines, such as interleukin IL-6 and tumor necrosis factor TNF-α (Fontana et al., 2007; Choi and Cohen, 2017). These mediators are secreted into the circulation and affect distant organs, increasing the risk of type 2 diabetes, atherosclerosis, cardiovascular disease, asthma (de Jong et al., 2002; Ji and Guo, 2019; Wallace et al., 2019), and several forms of cancer. Excessive adipose tissue also increases systemic TGF-β1, which switches fibroblasts to myofibroblasts and promotes fibrosis (Masszi et al., 2004). In addition to the above-mentioned secreted adipokines, extracellular vesicles (EVs) secreted by the adipose tissue, such as exosomes, also show the ability to regulate the pathophysiological level of the body (Li et al., 2015).

**FIGURE 1:**
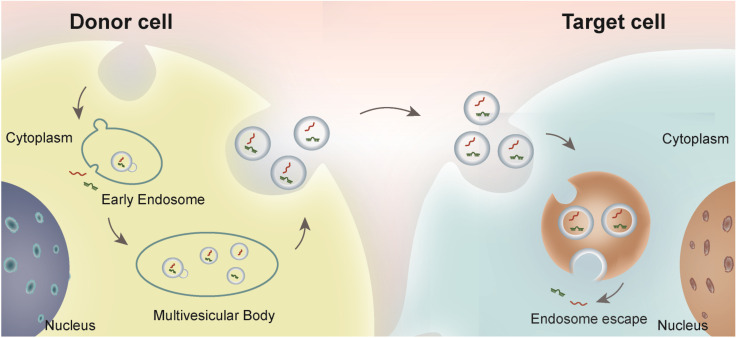
Schematic illustration of exosome biogenesis and intercellular communication. Exosomes, originated from the endosome in the donor cells, are secreted and endocytosed by the recipient cells either adjacent to or distal from the donor cells. The bioactive cargos could be escaped from the endosome in the recipient cells and play functional roles.

Extracellular vesicles refer to the collective name of micro vesicles with membrane structure that are actively secreted by cells (van Niel et al., 2018). According to physical and chemical properties and biochemical composition, EVs are mainly divided into exosomes, microvesicles and apoptotic bodies (Shao et al., 2018). Exosomes are nanoscale extracellular lipid bilayer vesicles with the diameter of 30–200 nm, which can be secreted by almost all types of cells (Wan et al., 2018; Wu et al., 2019). Exosome biogenesis usually starts from endosomal membrane invagination, forming the late endosomal multivesicular bodies (MVBs)/endosomes. During this process, cytosolic nucleic acids and proteins are incorporated into the lumen of MVBs. The exosomes are further secreted from the donor cells when the endosome fused with the plasma membrane (Wang et al., 2021). Almost all cells secrete EVs and play a pivotal role in intracellular communication and biological events. Exosomes contain various contents including proteins, lipids, and nucleic acids (DNA, RNA, and microRNA) (Lehmann et al., 2008; Guescini et al., 2010), which are widely involved in signal transduction and genetic information exchange. The exosome biogenesis process and intercellular communication are depicted in Figure 1. Due to the ubiquity of resources, stability in biological fluids, potent bioactivity, and continuous improvements in the quantitative and qualitative techniques, exosomes have become promising biomarkers and therapeutic carriers in clinical application (Zhang B. et al., 2019). For example, exosomes derived from tumor tissues can be used as biomarkers for cancer diagnosis. Exosomes are involved X-ray radiation and other canonical therapies in cancer treatment (Ahmadi and Rezaie, 2020). In addition, engineered exosomes could be used as vaccine or chemotherapy drug carrier (Tian et al., 2014). Beyond systemic delivery, exosomes could be also released by hydrogel for generation purposes.

Adipose tissue constitutes an important source of circulating exosomes, which can regulate gene expression in adjacent or distant tissues via the encapsulated miRNAs (Deregibus et al., 2007; Thomou et al., 2017; Burkova et al., 2018). Accumulating studies have found that exosomes released by adipose tissue may act importantly in maintaining the body’s homeostasis (Ji and Guo, 2019).

## Pathophysiological Roles of Adipose Tissue-Derived Exosomes

The homeostasis of the body is the result of the interaction between various tissues. As the largest secretory organ, adipose tissue releases various hormones and adipokines. The increased/enlarged adipose tissue in obese patients can cause various metabolic and cardiovascular complications (Labrecque et al., 2017). Recent studies identify adipose tissue derived exosomes as a type of intercellular communication mediator between adipose tissue and other tissues.

### Adipose Tissue-Derived Exosomes in Lipid Metabolism

Similar as the parental cells, adipose tissue derived exosomes also play essential role in lipid metabolism. Adipose tissue is rich in rich in enzymes related to the lipogenesis, such as acetyl-CoA carboxylase, glucose-6-phosphate dehydrogenase dehydrogenase, and fatty acid synthase. Consistently, adipose tissue derived exosomes are also enrich of these enzymes. Notably, under hypoxic conditions, the total amount of exosomes and the encapsulated enzymes by the adipose tissue are increased. In turn, these enzymes may affect lipogenic activity in the recipient cells (Sano et al., 2014). Moreover, abundant miR-450a-5p expression was found in adipose tissue derived exosomes, which can promote adipogenesis by inhibiting the expression of WNT1 inducible signaling pathway protein 2 (WISP2) (Zhang Y. et al., 2017).

Obesity is a recognized risk factor for atherosclerosis. Obesity is linked to coronary plaque progression and a high rate of cardiovascular disease events (Kahn et al., 2019). It has been reported that adipokines and cytokines secreted from accumulated VAT can affect lipid metabolism and have an impact on the formation of atherosclerosis (Martinez and Andriantsitohaina, 2017; Akbar et al., 2019; Scheja and Heeren, 2019). It has been found that adipose tissue derived exosomes from obese individuals can down-regulate expression of ABCA1 and ABCG1, which function importantly in cholesterol efflux, resulting in reduced cholesterol efflux, polarization of M1 and enhanced activation of NF-κB. Ultimately, adipose tissue derived exosomes from obese individuals accelerate the formation of macrophage foam cells, promote inflammation, and increase the risk of atherosclerosis (Xie et al., 2018).

### Adipose Tissue-Derived Exosomes in Regulation of Insulin Resistance

Adipose tissue functions importantly in insulin activity regulation. Specifically, adipose tissue in obesity dampens insulin sensitivity. Adipose tissue exosomes can directly interfere with insulin signaling in liver and muscle cells, which is related to o systemic insulin resistance (Kranendonk et al., 2014). For example, miR-27a derived from adipose tissue may inhibit PPARγ and its downstream genes, and play a key role in the occurrence of insulin resistance induced by obesity (Yu et al., 2018). The reduced miR-141-3p in adipose tissue exosomes of obese patients can significantly inhibit hepatocyte insulin sensitivity and glucose uptake (Dang et al., 2019). It has also been revealed that, exosomes from the adipose tissue macrophages (ATMs-Exos) in obese mice cause glucose tolerance and insulin resistance in lean mice via the miRNA cargos (Hubal et al., 2017). For example, miR-155 is highly expressed in the exosomes from obese ATMs-Exos. Exosomal miR-155 inhibits the target gene PPAR-γ together with several downstream PPAR-γ target genes in recipient liver cells, causing insulin resistance and abnormal glucose tolerance (Ying et al., 2017). Similarly, exosomal miR-29a causes insulin resistance via targeting PPAR-δ(Liu T. et al., 2019). Systemic inflammation is also considered as the leading cause of insulin resistance. Consistently, exosome-like vesicles (ELVs) released from adipose tissue are also found to play a role in macrophage activation and insulin resistance. ELVs are involved in the increased secretion of TNF-α and IL-6 by ATMs-Exos in obese mice. Mechanistically, ELVs induce insulin resistance induction at least partially through the TLR4/TRIF pathway (Deng et al., 2009).

### Adipose Tissue-Derived Exosomes in Regulation of Inflammation and Immunity

Inflammation in adipose tissue is the main cause of obesity-related metabolic complications. However, the molecular link between fat adipose tissue and inflammatory immune cells remains elusive. MiR-34a is a key component of exosomes secreted by adipocytes. Studies have shown that mature adipocytes secrete exosomes and transport miR-34a to macrophages, transmitting nutrient overload signals to macrophages residing in fat. In the recipient macrophages, miR-34a inhibits M2 polarization by inhibiting the expression of Krüppel-like factor 4 (Klf4), exacerbating systemic inflammation and contributing to the metabolic abnormalities caused by obesity (Pan et al., 2019). In contrast to the pro-inflammatory exosomes from the obese adipose tissue, ADSC-derived exosomes are found to promote M2 polarization in macrophages (Zhao H. et al., 2018).

Neutrophils are transient cells of the innate immune system and participate in defense against pathogens by generating reactive oxygen species (ROS). Exosomes isolated from interstitial stromal cells of human adipose tissue are found to increase the vitality of the neutrophils via the encapsulated protein cargos, resulting in improved immunity (Mahmoudi et al., 2019).

### Adipose Tissue-Derived Exosomes in Regulation of Other Systems

Besides the above regulation, accumulating evidence suggests that adipose tissue derived exosomes also function importantly in other systems. Exosomes of perivascular adipose tissue (PVAT) contain a large number of miRNAs. PVAT derived exosomes from the obese mice due to inflammation lead to vascular phenotypic transformation of the abdominal aorta in lean mice, which could be at least partially attributed to the increased expression of miR-221-3p. Mechanistically, miR-221-3p significantly enhances the proliferation and migration of vascular smooth muscle cell (VSMC) (Li X. et al., 2019).

A recent study profiled the proteomic content change of adipose tissue exosomes in obese women during pregnancy. Obese pregnant women’s exosomes are rich in proteins that associated with metabolism, such as mitochondrial dysfunction and SIRT signaling pathways (Jayabalan et al., 2019). Accordingly, the increased exosome release was positively correlated with the birth weight Z score, indicating that it may interact with the placenta and regulate fetal growth.

Obesity is linked with types of cancers. Recently Lu et al. (2017) revealed that the gluteal adipose exosome derived Hotair promote colon cancer cell proliferation via enhancing Wnt signal. In a pioneering study, Wei et al. (2020) revealed that high fat diet changed the miRNA profile of the visceral adipose exosomes, switching the exosome from anti-inflammatory to pro-inflammatory phenotype. In turn, these exosomes predisposed the intestine to inflammation via promoting macrophage M1 polarization. The study has established an exosomal pathway how obesity aggravates colitis, which well explained the strong association of obesity with the risk of colitis found in epidemic studies (Wei et al., 2020). We have also found that exosomes from the maternal adipose tissue could cross the placenta barrier, affecting the developmental embryos (Liu et al., 2021).

In addition, adipocytes are also one type of stromal cells of many different cancers. For example, hepatocellular carcinoma (HCC) is the main form of liver cancer and has shown an increased incidence and poor prognosis. Studies have shown that the effects of fatty exosomes promote tumor growth and reduce DNA damage by targeting deubiquitination-related USP7 via the exosomal circRNA (Zhang H. et al., 2019). In HCC patients with a body fat ratio, the expression of miR-23a/b in serum exosomes was significantly higher, compared with that in patients with low body fat. *In vitro* studies further suggest that exosomal miR-23a/b is most likely from fat cells, and it could promote the growth and migration of HCC cells when endocytosed (Liu Y. et al., 2019).

**FIGURE 2:**
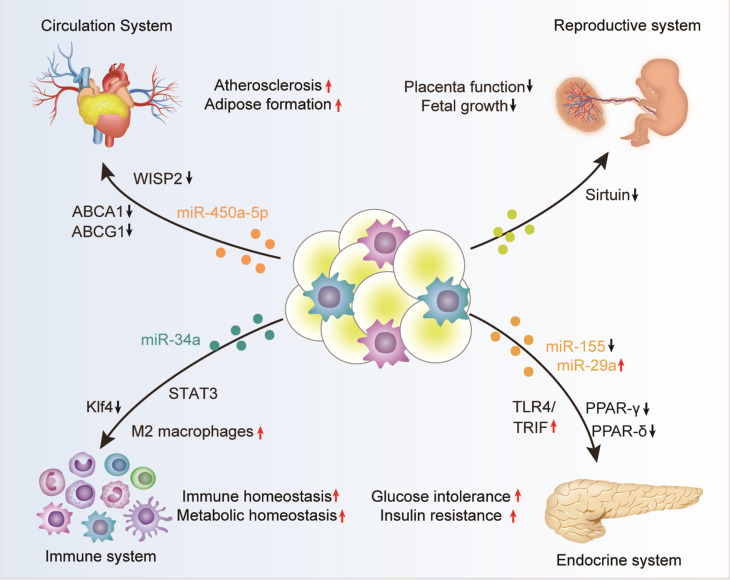
Adipose tissue-derived exosomes in the regulation of biological processes. Adipose tissue derived exosomes, either from the adipocytes, associated macrophages, or other stromal cells, can circulate into distant organs, such as the cardiovascular system, endocrine system, immune system, and reproductive system, where they can regulate the function of the recipient cells. The exosome-mediated crosstalk between adipose and other organs function importantly in many physiological and pathological contexts, especially obesity.

In summary, adipose tissue can regulate the function of multiple organs, such as the cardiovascular system, endocrine system, immune system, and reproductive system, in the form of exosomes (Table 1 and Figure 2). However, there are still too many to be revealed concerning the detailed function and the underlying mechanisms. With the deepening of research on the physiological and pathological effects of adipose tissue exosomes will help to better explore the mysteries of life and provide timely and effective clinical interventions.

**TABLE 1:** Adipose tissue-derived exosomes in the regulation of biological processes.

Source of exosomes	Biological function	Mechanisms	References
ATM derived exosomes	Insulin resistance	Inhibition of PPAR-γ and PPAR- δ; Upregulation of TLR4/TRIF	Deng et al., 2009; Ying et al., 2017; Liu T. et al., 2019
Gluteal fat with sedentary lifestyle	Cell proliferation	Hotair-Wnt axis	Lu et al., 2017
VAT exosomes	Colitis aggravation	miR-155-M1 polarization	Wei et al., 2020
WAT exosomes	Lipid metabolism regulation	Decrease of ABCA1, ABCG1 Suppression of WISP2	Sano et al., 2014; Xie et al., 2018; Zhang Y. et al., 2017
ADSC exosomes	Immune homeostasis	M2 polarization	Pan et al., 2019
WAT exosomes	Immune homeostasis	Inhibition of Klf4 expression	Zhao H. et al., 2018
WAT exosomes	Vascular remodeling	VSMC phenotype switch	Li X. et al., 2019
WAT exosomes	Placental barrier and fetal growth	Proteins related to cell metabolism	Jayabalan et al., 2019

## Therapeutic Implication of Adipose Tissue-Derived Exosomes

**FIGURE 3:**
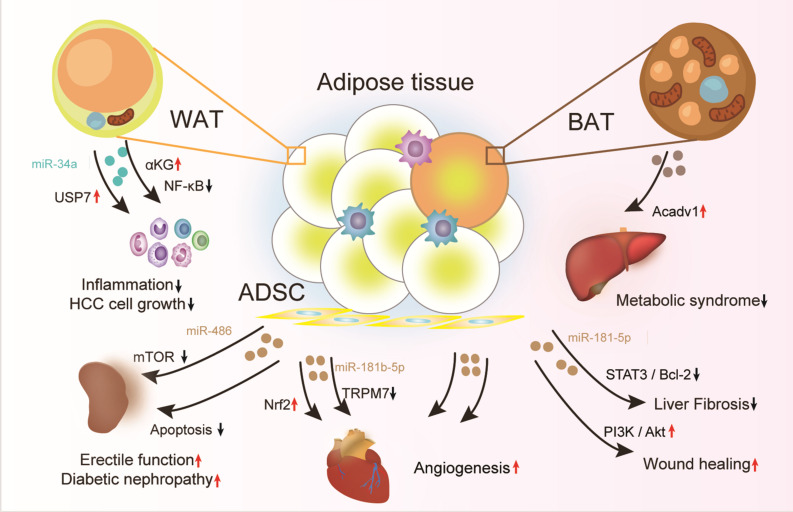
Therapeutic effects of adipose tissue-derived exosomes. Exosomes from healthy adipose tissue, specifically the adipose in lean, the brown adipocytes, and the ADSCs have great therapeutic potential for types of diseases, such as inflammation, metabolic syndrome, and endothelial dysfunction. Moreover, the adipose tissue should be engineered with chemical drugs or gene modification for production of exosomes with improved therapeutic effects.

The encapsulated RNA, miRNA, proteins and lipids in exosomes share similar profile as the parental cells. To this end, exosomes are considered as potential biomarkers and therapeutic drugs for many diseases (Figure 3 and Table 2).

**TABLE 2:** Therapeutic effects of adipose tissue-derived exosomes.

Source	Methods	Therapeutic implication	Mechanisms	References
ADSC Exosomes	Native exosomes	Promotion of wound healing	Upregulation of PI3K/Akt	Hu et al., 2016; Zhang W. et al., 2018
ADSC Exosomes	Native exosomes	Improvement of erectile dysfunction	Inhibition of apoptosis	Chen F. et al., 2017
ADSC Exosomes	Native exosomes	Promotation of angiogenesis	Promotion of cell proliferation and migration	Du et al., 2018
ADSC Exosomes	Native exosomes	Attenuation of diabetic nephropathy	Suppression of mTOR activation	Jin et al., 2019
ADSC Exosomes	Overexpressing of Nrf2	Promotation of angiogenesis	Inhibition of ROS and inflammatory cytokines	Li X. et al., 2018
ADSC Exosomes	Hypoxia-treated	Promotation of angiogenesis	Regulation of VEGF/VEGF-R	Han et al., 2019
ADSC Exosomes	Overexpression of miR-181-5p	Reduction of liver damage	Suppression of Stat3 and Bcl-2	Qu et al., 2017
ADSC Exosomes	Native exosomes	Promotation of angiogenesis	Regulation of miR-181b-5p/TRPM7	Yang et al., 2018
BAT Exosomes	Native exosomes	Reduction of obesity metabolic syndrome	Increase of liver energy metabolism	Zhou et al., 2020
Perivascular adipose tissue exosomes	Drug treatment	Regulation of vascular function	Regulation of NF-κB signaling	Brown et al., 2014; Zhao Q. et al., 2019
WAT Exosomes	Native exosomes	Decrease of food intake and weight	Regulation the expression of POMC	Gao et al., 2020
WAT Exosomes	Knock out circ-DB	Inhibition of HCC cell growth	Activation of the USP7/Cyclin A2 signaling pathway	Zhang H. et al., 2019

### Adipose Tissue-Derived Exosomes for Improvement of Endothelial Function

Adipose tissue-derived exosomes are considered to be an important regulator of cardiovascular health (Liang et al., 2016; Xu et al., 2019). Recently, PVAT is found to function importantly in the regulation of endothelial homeostasis (Schinzari et al., 2017). PVAT are the adipose tissue surrounding blood vessels, and factors secreted by adipocytes can easily target blood vessels. PVAT is considered a unique storage of adipose tissue, different from SAT and other VAT, especially in terms of regulation of vascular function (Brown et al., 2014). It has been revealed that exosomes derived from mangiferin stimulated PVAT ameliorate endothelial dysfunction, suggesting a potent benefit in the treatment of obesity-related cardiovascular diseases (Zhao Q. et al., 2019).

Adipose stem cells are a type of adult stem cells and have the potential to differentiate into other types of cells. Besides, adipose stem cells are of potent paracrine function. These features make them have broad prospects and potential in regenerative medicine. With the deepening of research, it has been proposed that the biological functions of adipose stem cells are closely related to their exosomes. ADSC-exos are considered to be a candidate drug for ischemic diseases (Zhao et al., 2017). Studies have shown that treatment of exosomes with from ADSCs overexpressing Nrf2 accelerate the healing of wound ulcers on the feet of diabetic rats, partially via enhanced angiogenesis (Li X. et al., 2018). Similarly, ADSC derived exosomes could promote angiogenesis via enhancing VEGF/VEGFR and/or miR-181b-5p/transient receptor potential melastatin 7 (TRPM7) axis (Yang et al., 2018; Han et al., 2019). The exosomes from miR-199-3p modified ADSCs promote the proliferation and migration of endothelial tip cells by down-regulating Sema3A (Du et al., 2018).

### Adipose Tissue-Derived Exosomes in Treatment of Metabolic Syndrome

As adipose tissue-derived exosomes are fundamental regulator of insulin resistance, the role of exosomes in the treatment of diabetes is now intensively studied (Zhang B. et al., 2019; Jin et al., 2020). Type 2 diabetes and obesity are diseases related to excess energy in the body. The abnormal interaction between the hypothalamus and adipose tissue is a key trigger for energy metabolism dysfunction. Adipocyte derived exosomes can regulate POMC expression *in vivo* and *in vitro* through the hypothalamic mTOR signal, thereby affecting the body’s energy intake (Gao et al., 2020). In addition, ADSCs-Exo suppresses mTOR activation, improving the diabetes-related complications (Jin et al., 2019).

The main function of BAT is to produce heat through UCP1 located on the inner mitochondrial membrane. Transplanting BAT from donor mice to recipient mice significantly improves the whole body energy metabolism, with the weight gain caused by high-fat diet prevented and insulin resistance reversed (Liu et al., 2013, 2015; Stanford et al., 2013). BAT can dissipate energy and can also regulate the metabolism of other organs through exosomal miRNA (Chen and Pfeifer, 2017). Very recently, we have shown that high-fat diet mice treated with BAT exosomes had reduced body weight and blood glucose levels, while serum exosomes treatment had such effect (Zhou et al., 2020). Mechanistically, the BAT-exosomes are enriched in functional mitochondrial components, such as ACADV1, which can be transferred to the recipient cells (Zhou et al., 2020). The survival rate is the big concern for brown adipocytes/adipose tissue transplantation. In contrast, there is no such concern for BAT-Exo treatment, while BAT-Exos have similar function as the BATs.

### Other Diagnostic and Therapeutic Implications of Adipose Tissue-Derived Exosomes

Besides the potential therapeutic effects on endothelial function and metabolism, exosomes could be also used in other contexts. For example, miR-181-5p in fat-derived mesenchymal stem cells (MSCs-Exo) can significantly down-regulate collagen I and vimentin in the liver to reduce liver damage (Qu et al., 2017). ASCs-Exos can optimize the characteristics of fibroblasts to achieve the purpose of promoting skin wound healing (Hu et al., 2016; Zhang W. et al., 2018) and improve erectile dysfunction in diabetic mice (Chen F. et al., 2017). Similarly, overexpression of miR-19b in VAT exosomes can reduce inflammation in placental cells (Liu et al., 2021). With the continuous deepening of related research, exosomes from adipose tissue or adipocytes or the precursor, are expected to widely used in different diseases.

## Conclusion and Perspectives

Exosomes produced by adipose tissue participate in the regulation of almost all physiological activities in the body. Besides the intensively studied miRNAs in exosomes, other bioactive molecules, such as lncRNA and circRNA, are worthy of further exploration. It is important to note that isolation of the exosomes from the adipose tissue is prerequisite for the profiling of the cargos. Since adipose tissue consists of types of cells. Isolation of the exosomes with adipocyte specific antibodies would be a promising and accurate strategy for the purpose of profiling adipocyte specific exosomes. In addition to the encapsulated cargos, the targeting specificity and the underlying mechanisms are also worthy of exploration. The adipose-derived exosomes should carry special exosomal membrane proteins, responsible for their special biodistribution.

The exosomes from healthy adipose tissue or the ADSCs have great therapeutic potential. Compared with the cells, exosome-based therapy has low immunogenicity, convenience for transportation and manufacture. Currently, there are multiple clinical trials exploring the therapeutic and diagnostic value of exosomes derived from adipose tissue or the stromal cells. The safety and efficiency of aerosol inhalation of the exosomes derived from allogenic adipose MSCs-Exo in severe patients with novel coronavirus pneumonia (NCP) has been performed recently (NCT04276987). In addition, a Phase 1/2 clinical trial to explore the safety and efficacy of the exosomes derived from allogenic adipose MSCs-Exos in the treatment of mild to moderate dementia due to Alzheimer’s disease, is also undergoing (NCT04388982). The effect of adipose derived stem cells exosomes as an adjunctive therapy to scaling and root planning in the treatment of periodontitis is also undergoing (NCT04270006). Beyond the therapeutic purposes, roles of exosomes from fat tissue in lean and obese patients are also explored in clinical trial (NCT04167722). The clinical trials will open an avenue for the diagnosis and therapy based on adipose derived exosomes.

Beyond the native exosomes, exosomes from adipose tissue could be engineered to encapsulate cargos of interest. CD9-HuR engineered exosomes have found to successfully enrich the overexpressed RNA cargos with AREs (Li Z. et al., 2019). We have shown that exosomes-mediated delivery of Ldlr mRNA can treat diseases in Ldlr^–/–^ mice (Li et al., 2021). Further engineering of exosomes from adipose MSCs-Exos would be a promising therapeutic strategy, especially when the wide source of adipose MSCs-Exo is considered.

The main limitations of in clinical application of adipose tissue-derived exosomes remain the resources and quality control among different batches. Isolation of the sufficient exosomes from the adipose tissue or establishment of the alternative resources is fundamental for potential clinical application. Techniques to ensure the exosome homogeneity during biogenesis, isolation, and quality control are also widely needed. The challenges could be solved when the fundamental procedures of exosome biogenesis and regulation are further clarified.

## Author Contributions

LY and GY designed the framework, analyzed the data, and revised the manuscript. YL, CW, and MW contributed to data collection and manuscript drafting. All authors agreed to submit the final version.

## Conflict of Interest

The authors declare that the research was conducted in the absence of any commercial or financial relationships that could be construed as a potential conflict of interest.
